# Eavesdropping Vulnerability and Countermeasure in Infrared Communication for IoT Devices

**DOI:** 10.3390/s21248207

**Published:** 2021-12-08

**Authors:** Minchul Kim, Taeweon Suh

**Affiliations:** 1 Department of Information Security, Korea University, 145 Anam-ro, Seongbuk-gu, Seoul 02841, Korea; betamc@korea.ac.kr; 2 Department of Computer Science and Engineering, Korea University, 145 Anam-ro, Seongbuk-gu, Seoul 02841, Korea

**Keywords:** infrared communication, eavesdropping, security, secure IoT system

## Abstract

Infrared (IR) communication is one of the wireless communication methods mainly used to manipulate consumer electronics devices. Traditional IR devices support only simple operations such as changing TV channels. These days, consumer electronic devices such as smart TV are connected to the internet with the introduction of IoT. Thus, the user’s sensitive information such as credit card number and/or personal information could be entered with the IR remote. This situation raises a new problem. Since TV and the set-top box are visual media, these devices can be used to control and/or monitor other IoT devices at home. Therefore, personal information can be exposed to eavesdroppers. In this paper, we experimented with the IR devices’ reception sensitivity using remotes. These experiments were performed to measure the IR reception sensitivity in terms of distance and position between the device and the remote. According to our experiments, the transmission distance of the IR remote signal is more than 20 m. The experiments also revealed that curtains do not block infrared rays. Consequently, eavesdropping is possible to steal the user’s sensitive information. This paper proposes a simple, practical, and cost-effective countermeasure against eavesdropping, which does not impose any burden on users. Basically, encryption is used to prevent the eavesdropping. The encryption key is created by recycling a timer inside the microcontroller typically integrated in a remote. The key is regenerated whenever the power button on a remote is pressed, providing the limited lifecycle of the key. The evaluation indicates that the XOR-based encryption is practical and effective in terms of the processing time and cost.

## 1. Introduction

Infrared (IR) communication has been used to wirelessly control electronic devices such as TVs, air conditioners, audio, beam projectors, and healthcare gadgets. Some works utilize the IR module to control home appliances by recognizing human gestures with wearables such as armbands [1], or gloves [2], or other sensing devices [3]. Some research works also use the IR device as a medium to take commands from smartphones and send those to home electronics [4,5,6,7,8]. The IR communication is also used to operate special devices such as robots [9,10]. For communication, it is required to have Infrared Emitting Diode (IRED) on the transmission side and IR photodiode on the reception side. The hardware components on the transmitter and receiver are cheaper than the ones in other communications such as Wi-Fi, Zigbee, and Bluetooth, and the power consumption is low [11]. Since the IR communication uses light, it does not collide with the radio signal such as Wi-Fi, Zigbee, Bluetooth, and 5G. The IR channel can be used freely because it is unregulated [11].

The IR communication has several shortcomings. First, it can be disturbed by natural light, artificial light, and/or atmospheric dust. Another drawback is that that the communication distance is short. The last one is that the reception angle is narrow, meaning that the transmitter and the receiver should better face each other for seamless communication. If the angle between the transmitter and the receiver is not within a certain range, the communication is not stable or may not even be possible [11,12,13,14,15]. To overcome these limitations in practice, electronic devices are designed with two and more IREDs and/or reflector. This enables a farther and wider communication for user’s convenience. Unfortunately, it could incur security breaches.

Traditionally, only simple operations have been supported with the IR remote controller (referred to as remote hereafter). For example, TV can be turned on or off, and TV channels can be changed with a remote. These days, complex and intelligent operations are supported in electronic devices, especially with the introduction of IoTs. For example, smart TVs are equipped with an operating system such as Android [16]. These TVs are connected to the internet and provide various services requiring personal information. To purchase goods and/or watch content, the private and sensitive information such as credit card number, resident ID, or ID/PW should be entered with the IR remote. Smart TV can also serve as a bridge for IoT devices used at home. Thus, users can operate and/or monitor the IoT devices through the TV.

**Figure 1:**
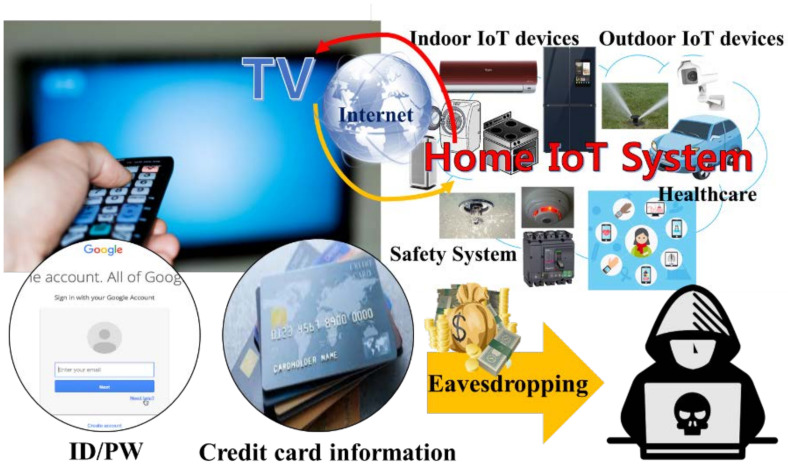
Eavesdropping threat when using infrared communication.

Figure 1 shows the eavesdropping threats using the IR. Eavesdropping is the act of secretly listening to the communication of others without consent, in order to gather information. The eavesdropper can be placed outside the home or company buildings. Thus, the users could be exposed to an environment where sensitive information can be leaked by eavesdroppers. The IR eavesdropping can happen in several other places. For example, hospitals have a variety of life-supporting devices using radio communication and are strictly managed to prevent communication collisions. Some research studies [17,18] show that light such as infrared can be used to send information related to a patient, not disturbing the radio communication. While using the IoT device, it would be a huge threat if patients’ sensitive health information such as patients’ disease [19] is leaked and/or tampered with. Another example is the automatic payment system in the highway tollgate [20]. The attacker can lower his/her toll by manipulating the entry place.

IR communication is open to the public. Anyone with a simple system with a microcontroller unit (MCU), IRED, and IR photodiode can collect data and/or operate a device. This paper demonstrates the eavesdropping threat and proposes an inexpensive countermeasure for security. The countermeasure means an action taken to counter or offset the threat.

This paper is organized as follows: Section 2 summarizes the related work; Section 3 discusses background information; Section 4 reports the IR reception sensitivity; and Section 5 demonstrates the eavesdropping threat. Section 6 presents a countermeasure for security and Section 7 concludes this paper.

## 2. Related Works

Security is one of the big concerns in the IoT domain due in part to the low-cost requirement. In particular, personal information and privacy should be protected and guaranteed because many IoT devices tend to be intertwined with personal and daily lives. There are various research efforts for IoT security. Rihab et al. [21] use facial and/or voice recognition as a method of preventing unauthorized access to IoT. Mamun et al. [22] worked on cryptography applicable to IoT by analyzing lightweight symmetric encryption algorithms such as CLEFIA and TRIVIUM. Pietro et al. [23] statically analyzed web applications used in IoT. They discussed top 10 vulnerabilities in The Open Web Application Security Project (IoT 2018) and how these can be exploited. Xinyu et al. [24] proposed a Secure Wi-Fi IoT Communication Router (SecWIR) framework designed on top of home Wi-Fi routers. This work was motivated by the fact that some router devices have either no security protocols deployed, or have problematic security protocol implementations. SecWIR claims to provide a secure IoT communication capability to home IoT devices.

There are two research efforts regarding security issues with IR communication. Zheng et al. [25] developed an IR electronic gadget inside a keyboard to steal sensitive information and control IoT devices autonomously. It was designed to attack IoT devices using IR from a remote site. The suggested countermeasures focus on the detection methods: sniffing the IR signal using the IR receiver tool and measuring the power consumption. Zhen et al. [15] designed an IoT system interconnecting legacy IR devices. They aimed to securely control these IR devices over the internet with a smartphone. The Raspberry Pi-based system equipped with a Wi-Fi module and IR transceiver acts as a bridge between the smartphone and the legacy IR devices. The smartphone is connected to clouds using the TLS protocol. The paper described threats against the legacy IR devices such as replay attack, brute-force attack, and drone attack. It demonstrated that the custom-built IR transmitter is able to reach as far as 47.31 m. It also showed that a drone flying outside a building can turn a TV on and off inside a conference room. To prevent such attacks, the paper stressed the importance of secure IR communication with encryption. However, the authors were concerned about the hardware cost and IR frame transmission delay that comes with the encryption. Our paper is different from Refs. [15,25] in that we propose an inexpensive and close-to-zero-delay scheme for secure IR transmission.

In addition to IR remotes, the RF technology is gaining popularity these days in remote controllers. The RF remote typically uses 2.4 GHz for communication. IR and RF technologies adopted in the remote have pros and cons [26]. Table 1 shows the differences between these two technologies. The transmission distance of IR is known to be roughly up to 10 m, whereas it is up to 50 m with RF remotes. The IR signal is blocked by objects and/or walls, whereas the radio signal can pass through those obstacles. This means that the eavesdropping risk is much higher with RF remotes. IR communication is not restricted by international regulations, so it can be used freely. On the other hand, RF communication should follow the regulations of international organizations such as the European Telecommunication Standards Institute (ETSI). In the case of RF remotes, there is also a risk of conflicts with other wireless technologies such as Bluetooth or Wi-Fi [27] since they use the same 2.4 GHz frequency band. The hardware cost of the RF remote is typically more expensive than the one for IR remote. 

## 3. Background

### 3.1. Infrared Spectrum

**Figure 2:**
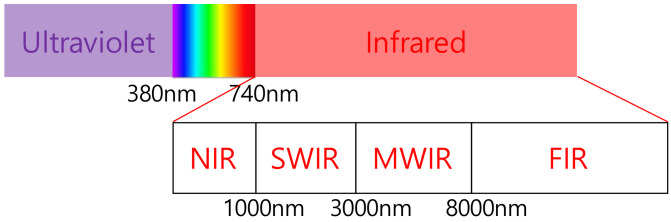
Infrared light spectrum.

Figure 2 shows the infrared spectrum with a wavelength of 740 nm or more outside the visible light range. Infrared rays are classified into near infrared (NIR), short-wave infrared (SWIR), mid-wave infrared (MWIR), and far infrared (FIR) [29,30]. The IR frequency is not reserved and thus can be used freely. The remote utilizes NIR, of which the wavelength ranges from 740 nm to 1000 nm [31,32]. The IR communication is also possible in the SWIR [13,33]. The naked eyes are not able to see the infrared.

### 3.2. Communication Protocols Using Infrared

**Figure 3:**
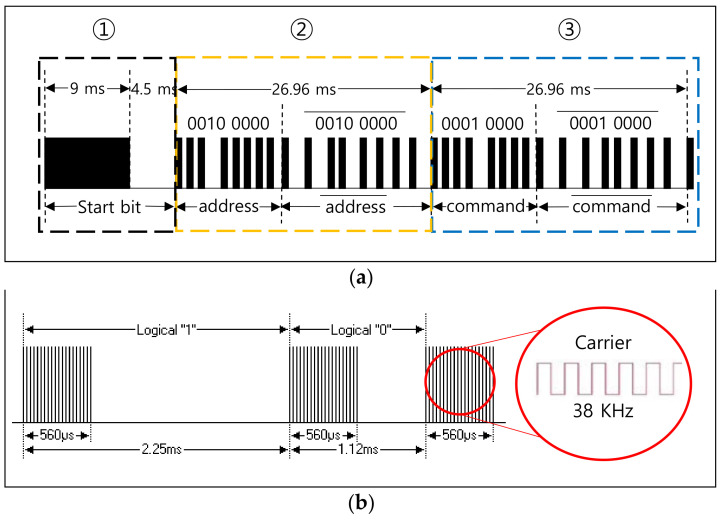
NEC protocol. (**a**) Frame format (example with power button of LG TV remote). (**b**) ‘0′ and ‘1′representation.

There are several communication protocols using IR such as Aiwa, BoseWave, Denon, Dish, JVC, Lego, LG, Mitsubishi, NEC, Panasonic, RC5, Samsung, Sanyo, Sharp, and Sony [28]. The NEC [15,28,34,35] is one of the widely used protocols. Figure 3 shows the frame format of the NEC protocol, which is designed to transmit a 32-bit in one frame. As shown in Figure 3a, a frame is composed of 1-bit start, 8-bit address, and 8-bit command. The start bit (①) is required to identify the beginning of the frame because it is based on asynchronous communication. The 8-bit address (②) is used to identify the device and the 8-bit command (③) is used to deliver the pressed key information in the remote. The inverted address and command are appended as shown, to verify the received information. Figure 3b shows the way to transmit the frame data, which is modulated with 38 KHz carrier. The logical ‘1′ is represented with 560 μs assertion and 1690 μs deassertion. The logical ‘0′ is represented with 560 μs assertion and 560 μs deassertion.

## 4. IR Reception Sensitivity Experiments

**Figure 4:**
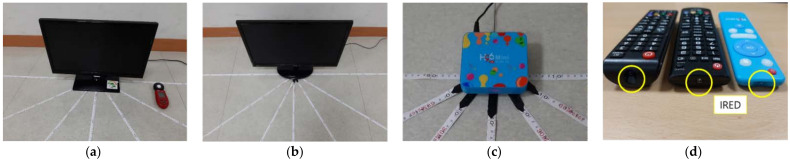
Experimental gadgets. (**a**) Samsung TV. (**b**) LG TV. (**c**) Set-top box. (**d**) Remotes.

This section measures the reception sensitivity of IR modules integrated into consumer electronic devices. The IR reception sensitivity refers to the distance, position, and rotation to which electronic devices can respond to the IR transmitter. Figure 4 shows experimental gadgets including Samsung TV, LG TV, set-top box, and each device’s remote.

**Figure 5:**
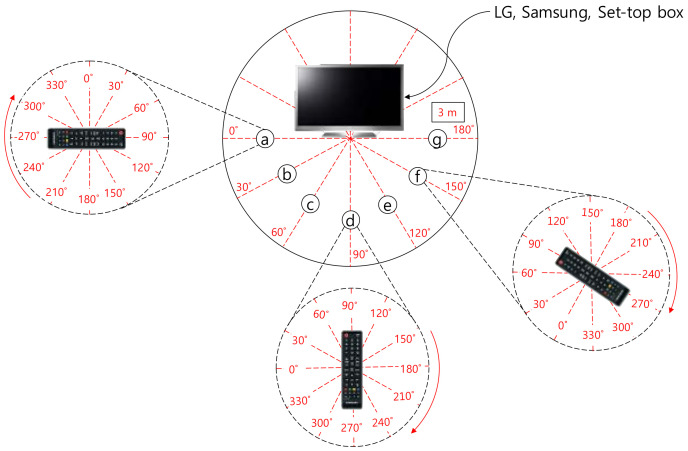
Remote’s positions (ⓐ~ⓖ) and angles at each position.

We have first measured the reception sensitivity according to positions and rotations. Figure 5 shows the experimental environment where the remote’s position was changed by 30°, marked as ⓐ~ⓖ. The remote’s angle was also changed by 30° in each position. The distance between the TV and the remote was fixed to 3 m in this experiment. The IR communication is known to be affected by external illumination [11,12,15]. Thus, the experiments were conducted under two different ambient lights in an empty room with a size of 8 m × 7 m; dark environment with 0 lx and bright environment with roughly 700 lx. The remote was pressed 10 times in each rotation at each position of ⓐ~ⓖ.

**Figure 6:**
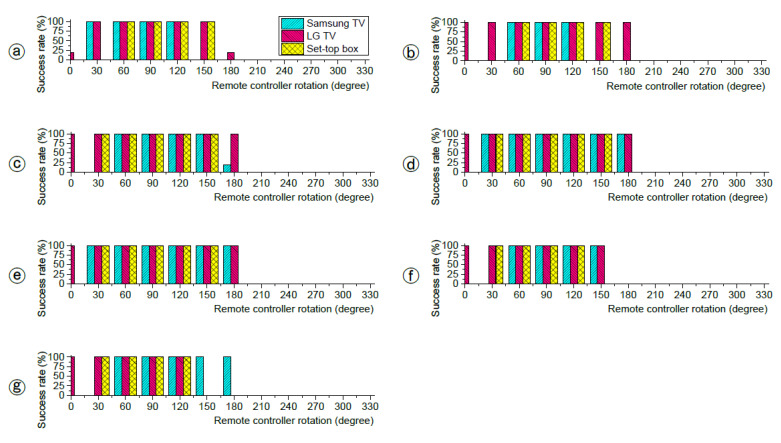
IR reception success rate according to angles.

Figure 6 shows the experiment results. The remote was pressed 10 times in each rotation at each position of ⓐ~ⓖ. The x-axis represents the remote’s rotated angles. The 90° means that the remote was directly pointing at the devices (TVs or set-top box). The y-axis is the success rate at which a certain number of trials were responded to by the devices.

Regardless of the positions, the devices responded when the range of angles was 60°~120°. This includes the angle at which the remote was directly pointing at the devices. However, the devices did not respond when the angle range was 210°~330° in all positions. The devices responded to a wider angle in ⓒ, ⓓ, and ⓔ positions than the others. For example, in the ⓓ position, the Samsung TV responded with the remote angled to 30°~180°. As the remote positioned further away from the center (ⓓ), the response tended to be unstable. For example, the Samsung TV responded when the angle was 180° in the position ⓖ, but did not respond in the position ⓕ.

**Figure 7:**
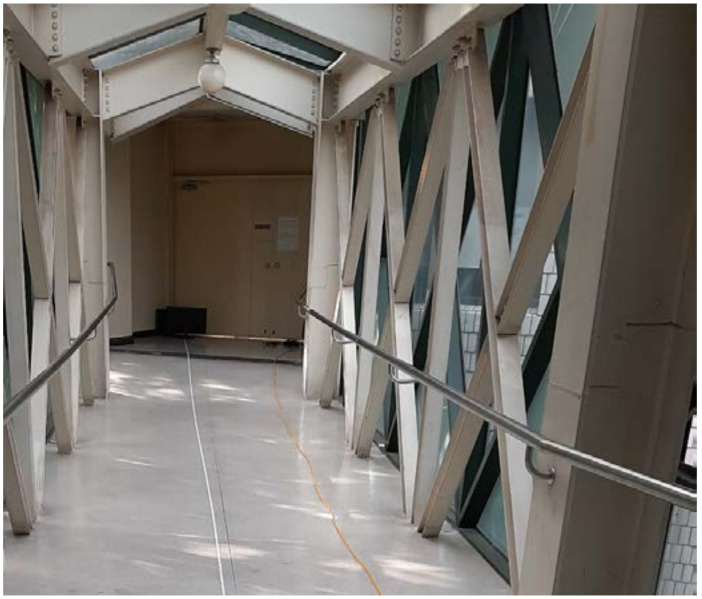
Environment measuring IR reception distance.

**Figure 8:**
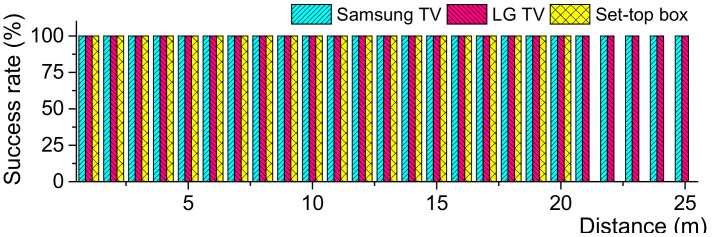
Response success rate according to distances.

In the second experiment, we have measured the distance from which the devices respond as the IR remote moves away. The experiment on distance is important because it indicates how far the attack would be possible from. Figure 7 shows the experiment environment where the distance can be as far as 25 m. The ambient light is roughly 3170 lx under sunlight. The remote was also pressed 10 times at each distance from 1 m to 25 m. Figure 8 shows the experiment results. The Samsung and LG TVs responded up until the maximum distance of 25 m. However, the set-top box responded from a distance of up to 20 m.

## 5. Eavesdropping Experiments

**Figure 9:**
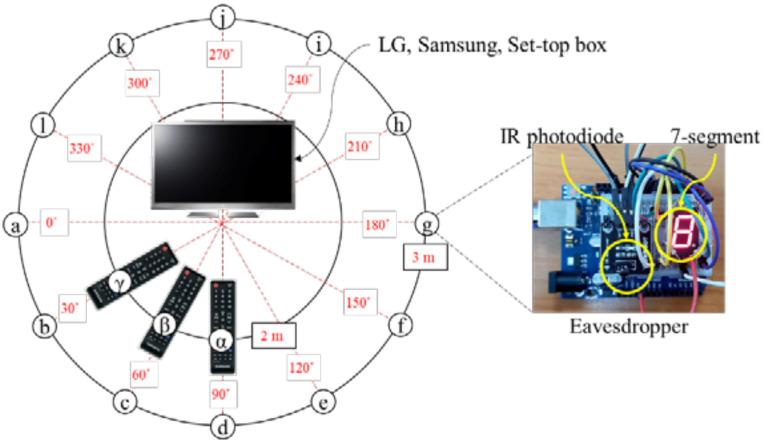
Eavesdropping experimental environment: eavesdropper’s position (ⓐ~ⓛ) and remote’s position (α, β, and γ).

In this section, we performed experiments on eavesdropping on the IR frames from the remotes. Figure 9 shows the experimental environment where eavesdropper’s positions are marked as ⓐ~ⓛ and remotes are located at α, β, and γ. The experiment was performed in a 7 m × 12 m empty room. The eavesdropper was designed using Arduino [36], an IR photodiode, and a 7-segment. The 12 eavesdroppers were positioned 3 m away from the TVs or the set-top box at ⓐ~ⓛ. The remotes were pressed at three different positions (α, β, and γ).

Table 2 shows the IR protocols and keypad mapping that the remotes utilize. Samsung uses its own protocol, and LG and the set-top box implement the NEC protocol. The difference in the protocols is the format in the address and command sections shown in Figure 3a. The 7-segment on the eavesdropper was used to check the received data visually. For example, the eavesdropper displays a ‘1′ on the 7-segment when the ‘On/Off’ keypad input from the Samsung remote is received. The experiments were repeated 10 times by alternately pressing the two buttons on each remote.

**Figure 10:**
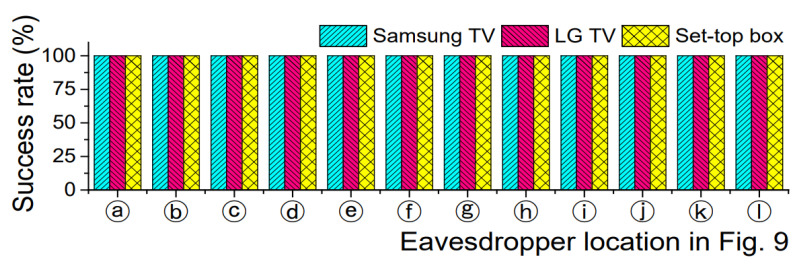
Eavesdropping experiment results in α, β, and γ positions.

Figure 10 shows the experiment results. Regardless of the remote’s positions (α, β, γ), all the eavesdroppers surprisingly collected the correct data even from behind the remote. The eavesdropping demonstration is available at https://youtu.be/VXxg-IvlqVU (Video S1: accessed on 8 December 2021).

**Figure 11:**
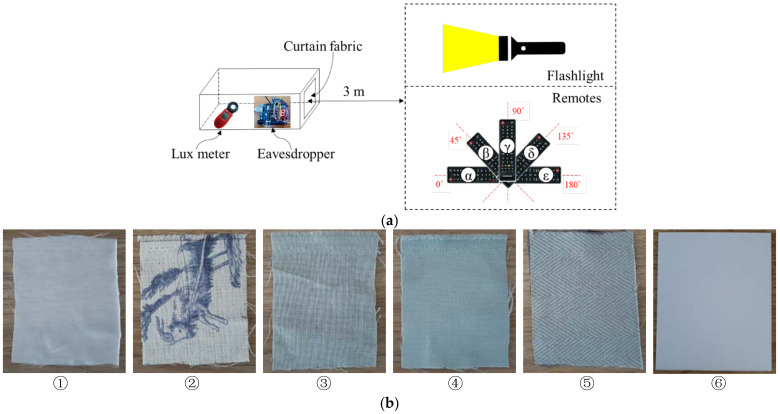
IR eavesdropping experimental setup with curtains and hardcover paper. (**a**) Measurement setup. (**b**) Curtain fabrics (①~⑤) with different light transmission characteristics and hardcover paper (⑥).

We further experimented with the possibility of collecting keypad inputs from outside. Figure 11 shows the experimental environment where the virtual home surroundings were set up. It is a virtual setup resembling an eavesdropper located behind the windows with curtains at a certain home. In the experiments, the remote points to various angles as shown in Figure 11a. The experimental environment was located in an empty space of 3.7 m × 2.45 m, and the ambient light was roughly 700 lx. The brightness of the light source was 9800 lx. People use curtains or blinds on windows at home. The curtains play a role in both blocking light and protecting privacy. We used diverse curtains (①~⑤) and hardcover paper (⑥) shown in Figure 11b, exhibiting different light transmission characteristics.

Table 3 shows the result of the light transmittance measurement depending on the curtain fabrics. The fabric ① does not block any light whereas the fabric ⑤ and hardcover paper ⑥ completely block the light.

**Figure 12:**
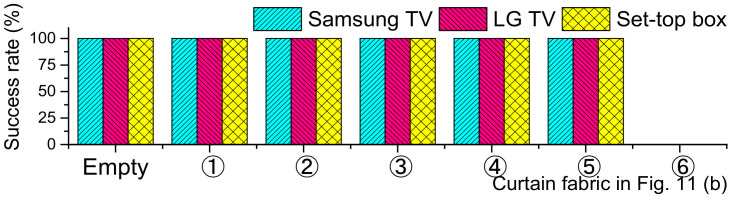
Success rate of eavesdropping behind curtain fabrics.

Figure 12 shows the success rate of eavesdropping by pressing the remote 10 times. Surprisingly, the eavesdropper successfully collected all keypad inputs from all angles (α~ε) even with the fabric ⑤, which completely blocks light.

**Figure 13:**
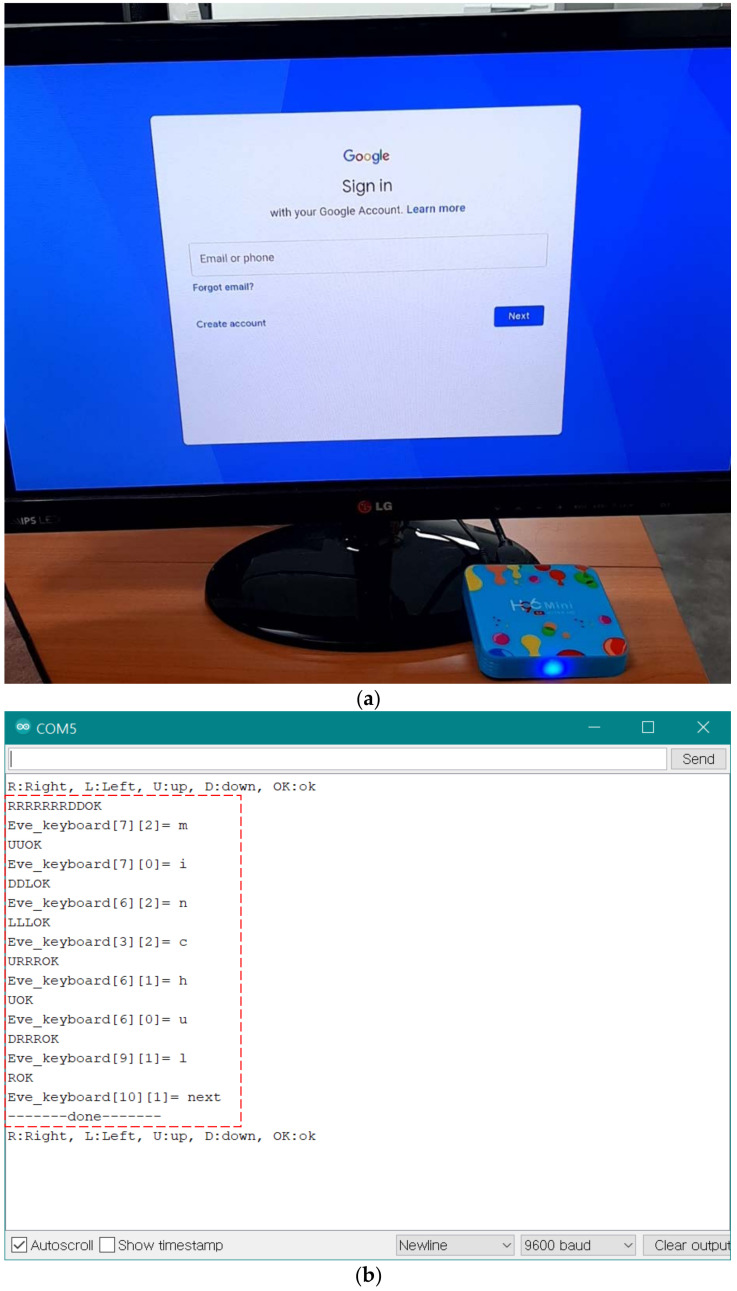
Risk of exposing sensitive information. (**a**) E-mail access with set-top box. (**b**) Picture of eavesdropping ID and password.

Simply eavesdropping on the TV remote may not seem dangerous because the changing of channels or increasing volume has nothing to do with personal information. However, a problem arises when a user authenticates himself/herself or places an order by accessing the internet via TV. These days, TVs typically provide network capability, as all the electronic devices are connected in the IoT era. For example, Figure 13a shows a picture accessing the e-mail account via the set-top box. The user enters the ID and password to authenticate himself/herself. Figure 13b shows a picture of the theft of a user’s ID and password using a designed eavesdropper. The exposed sensitive data, ID, and password can be used for exploitation.

## 6. Countermeasure

This section proposes a countermeasure to prevent the eavesdropping. The countermeasure should be simple and effective since the remote is usually used in consumer electronic devices where the cost is an important metric to consider. Furthermore, in the consumer electronics sectors, it is also required to minimize the customers’ involvement yet still provide a secure mechanism. Basically, a simple encryption can be used to effectively block the eavesdropping. In a typical communication system with cryptography, the key is shared between two parties in a handshaking manner. However, in the IR system, the key cannot be shared in the challenge-response manner at the beginning because the IR communication is based on the simplex; one party should pass the key to the other party. The key itself can be generated with two different methods: the user-generated and the device-generated methods. The former means that a user sets a password (key) by pushing buttons on the remote when using the remote for the first time. However, it is burdensome because the user must remember the password all the time and the password must be set again when replacing the battery in the remote or when using a new remote. In addition, there is a risk of password leakage from social engineering because people often use a meaningful sequence of numbers such as birthdays or phone numbers. The device-generated method automatically generates a key from the device. A key can be generated from the free-running timer in an MCU [37,38,39] typically integrated inside remotes. We use the latter for the key creation. The life cycle of the key is also an important issue because the key could be leaked eventually if the same key is used all the time. Thus, the key should be changed after a certain period of time. This paper takes an aggressive approach to security and generates a new key each time the power button in a remote is pressed.

**Figure 14:**
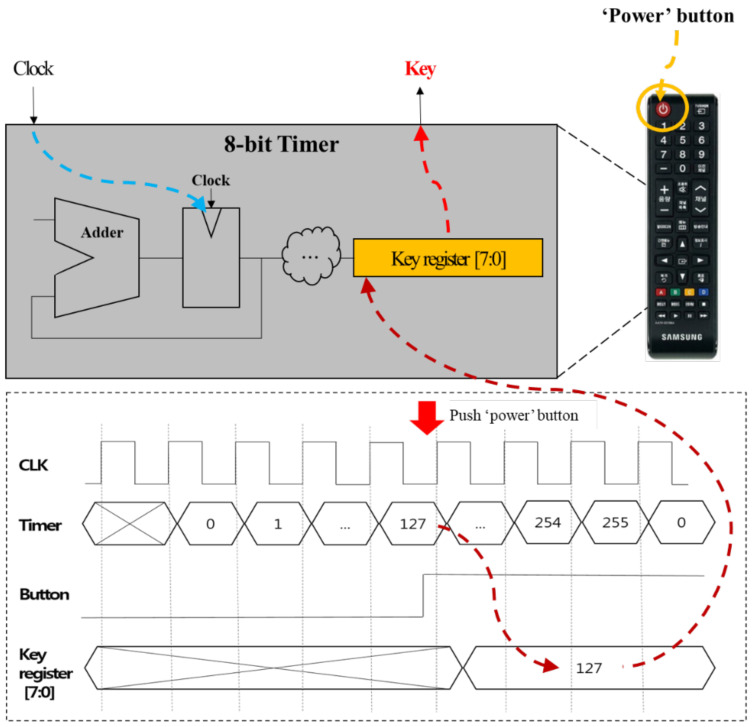
Key generator using 8-bit timer.

Figure 14 shows the proposed hardware key generator using an 8-bit timer inside a remote. The timer value is incremented every rising-edge of the clock and wraps around to 0 when reaching a maximum (255). This process is repeated continuously. When the power button is pressed on the remote, the timer value at that time is stored in the key register. The stored value is used as a disposable session key. This key is used until the TV is turned off. When the TV is turned on the next time, a new random key is created in the same way.

**Figure 15:**
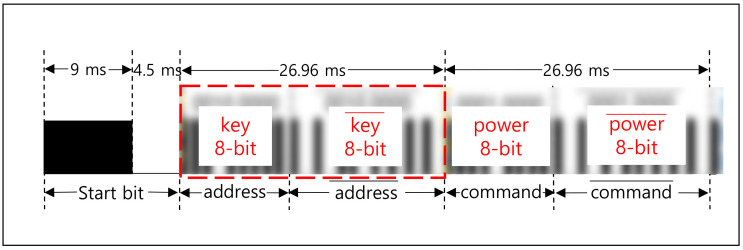
Key transmission via address field in NEC protocol.

Figure 15 shows the key transmission scheme. Many electronic devices utilize the NEC protocol for the IR communication. The address and command values are customized and assigned by the manufacturer following the format explained in Section 3. The address is typically fixed for each electronic device. For example, the experimented Samsung TV, LG TV, and set-top box use 0 × E0, 0 × 20, and 0 × 01, respectively, as shown in Table 2. We use the address field to transmit the key only when the power button is pressed as depicted in Figure 15. In other words, when the value for the power button is in the command field, the address field contains the key.

**Figure 16:**
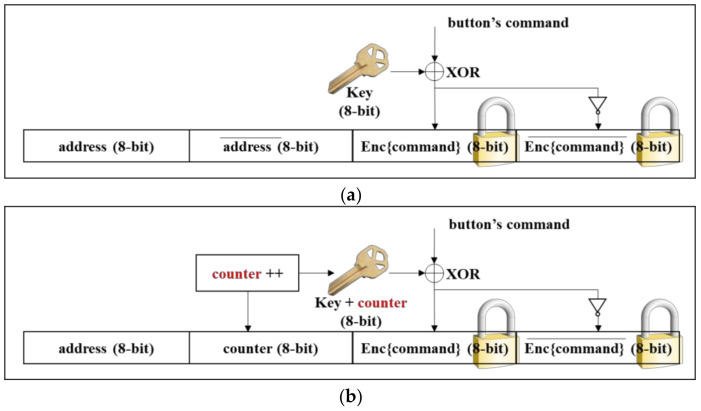
Proposed encryption schemes. (**a**) Command encryption using the key. (**b**) Command encryption using the key and counter.

Figure 16a shows the encryption scheme with the key. The command assigned to each button (except the power button) is encrypted using an XOR operation with the key, as in Equation (1). The XOR is a simple operation. Thus, the hardware cost is low and the processing time is negligible. Code 1 shows a pseudocode for encrypting an 8-bit command with a key and creating an IR packet.

Enc(8-bit command, 8-bit key) = 8-bit command ⨁ 8-bit key,(1)

One problem with this scheme is that the encrypted command during normal operation can be the same as the command for the power button, in which case the device is turned off. To avoid this problem, we impose a restriction that the device’s address is not allowed to be used as a key. This means that when the device’s address is delivered, the command should be decrypted with XOR on the receiver side. The strength of the encryption depends on the 8-bit key for the scheme in Figure 16a.
**Code 1**. Command encryption using the key and IR packing encoding1:  Procedure2:  3:    key = 8-bit key;4:    addr = 8-bit device_address;5:     cmd = 8-bit command;6:    enc_cmd = key ^ cmd; // encrypted command with XOR7:     8:     // create a 32-bit frame data according to NEC protocol9:    16-bit_ext_addr = (addr << 8) + ~addr;10:    16-bit_ext_cmd = (enc_cmd << 8) + ~enc_cmd;11:    32-bit_addr_cmd = (16-bit_ext_addr << 16) + 16-bit_ext_cmd;12: 13:   // Send the 32-bit frame via IR14:    IR_send (32-bit_addr_cmd)15: 16: End procedure

The simple XOR scheme using a fixed key could be easily deciphered by eavesdroppers with the brute-force attack. This is because the same key is used until the device is turned off. To cope with the problem, we introduce a counter so that the encrypted command becomes a different value for the same button. Figure 16b shows the proposed encryption scheme using a key and an 8-bit counter. The command is encrypted with the same XOR operation yet with ‘*key* + *counter*’ as in Equation (2). Code 2 shows a pseudocode for encrypting an 8-bit command with a key and creating an IR packet.

Enc(8-bit command, 8-bit key) = 8-bit command ⨁ ((8-bit key + 8-bit counter value) mod 256),(2)

**Figure 17:**
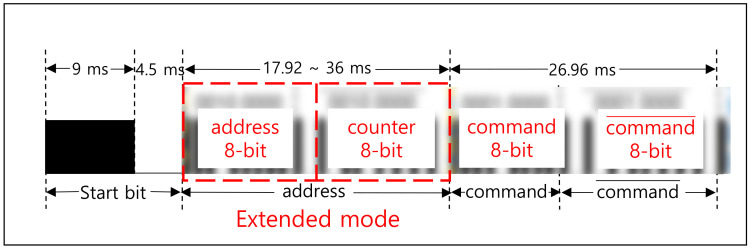
The ‘8-bit counter’ transmission using extended mode in NEC protocol.

The counter is incremented when the button on the remote is pressed each time, like the counter mode in encryption [40]. This means that the counter value should be passed to the receiver side for decryption. To transmit both the 8-bit counter and an 8-bit address, the extended mode [35] in the NEC protocol can be utilized. Figure 17 shows the frame format in the extended mode where a 16-bit address is transmitted instead of an 8-bit address. We use the 16-bit address field to transmit both the 8-bit address and 8-bit counter, as shown in Figure 17. In this scheme, the encryption strength is based on ‘*key* + *counter*’, which is also an 8-bit. However, eavesdroppers should guess the 8-bit value every time the remote button is pressed. For example, the probability of correctly extracting the user ID in Figure 13b is (1/256)^7^ because seven buttons were pressed.
**Code 2**. Command encryption using the key and counter1:  Procedure2:  3:    // random based on free-running timer4:    counter = 8-bit random_number;5:    key = 8-bit key;6:    addr = 8-bit device_address;7:     cmd = 8-bit command;8:     key = key + counter;9:    enc_cmd = key ^ cmd; // encrypted command with XOR10:     11:     // create a 32-bit frame data according to NEC protocol12:     16-bit_ext_addr = (addr << 8) + counter; 13:     16-bit_ext_cmd = (enc_cmd << 8) + ~enc_cmd;14:     32-bit_addr_cmd = (16-bit_ext_addr << 16) + 16-bit_ext_cmd;15:     16:    // Send the 32-bit frame via IR17:     IR_send (32-bit_addr_cmd)18: 19:    counter++;20: End procedure

**Figure 18:**
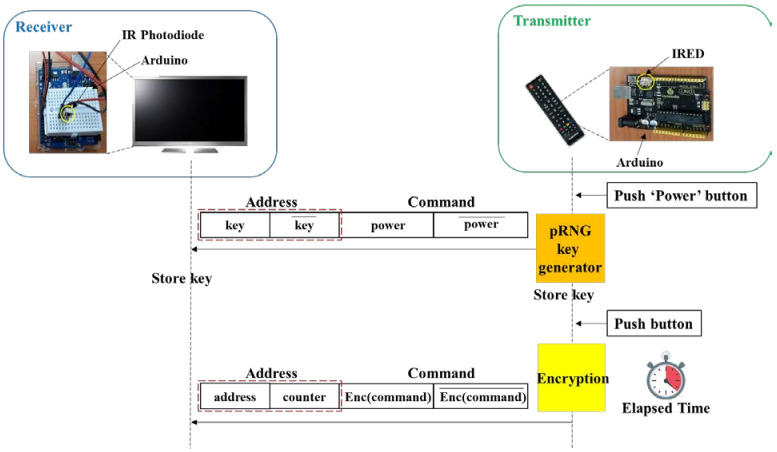
Experimental setup for performance evaluation.

Figure 18 shows the experimental setup for the evaluation of the countermeasure. A remote was designed with IRED and an inexpensive MCU called Arduino, and the receiver was designed with an IR photodiode and an Arduino. The key was created using pRNG [41], which is a pseudo-random number generator based on an interruption raised by a watchdog timer.

**Figure 19:**
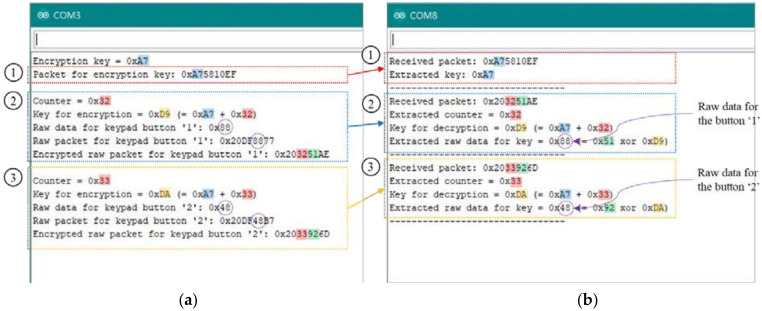
Log messages from transmitter and receiver in Figure 18: raw, encrypted, and decrypted data for keypad buttons ‘1′ and ‘2′ with the encryption key and counter values. (**a**) Transmitter (in Figure 18)’s log messages. (**b**) Receiver (in Figure 18)’s log messages.

Figure 19 shows the experimental results showing log messages from the IR transmitter and receiver. It shows the raw, encrypted, and decrypted data for the keypad buttons ‘1′ and ‘2′. When the power button is pressed, the transmitter sends the encryption key randomly taken from a counter and the receiver recovers it from the IR packet (① in Figure 19). ② and ③ show the processed and recovered data for the keypad buttons ‘1′ and ‘2′, respectively. The raw data for the buttons ‘1′ and ‘2′ are 0 × 88 and 0 × 48, respectively. The encryption for the button ‘1′ is performed with the encryption key (0 × A7) and a random counter value (0 × 32). The encryption for the button ‘2′ is performed with the encryption key (0 × A7) and the incremented counter value (0 × 33). The receiver recovers the raw data (0 × 88 and 0 × 48) from the received IR packets with the same XOR operation.

To estimate the overhead associated with the proposed method, we have measured the average elapsed time with and without encryption. The elapsed time is the time duration during which the frame information is created upon pressing a remote button. It was measured 10 times and averaged. The average elapsed time is 27,600.80 µs and 27,898.80 µs without and with encryption, respectively. The difference is negligible because a simple XOR operation is used in the countermeasure. The hardware cost of designing a remote with the proposed scheme is also negligible. The proposed scheme in Figure 16b requires 8 XOR gates for encryption and two 8-bit flip-flops for storing an 8-bit key and an 8-bit counter value.

## 7. Conclusions

In this paper, we demonstrated the eavesdropping threat with the IR communication and proposed a countermeasure. These days, consumer electronic devices such as smart TVs are connected to the internet for the IoT capability. The IR is an attractive wireless communication method because it is designed inexpensively and does not interfere with the radio signals. In the reception sensitivity experiments, Samsung and LG TV were responded to from farther than 25 m. This means eavesdropping is feasible within a wide range of the area. According to the eavesdropping experiments, the curtains were not able to block the eavesdropping. The proposed countermeasure uses a simple XOR operation with a key. The remote operation time with and without encryption differed by only roughly 300 µs in the experiment. The hardware for encryption requires only eight XOR gates and two 8-bit flip-flops for the key and counter. The proposed solution is simple enough to be implemented in consumer electronic products and does not require the user’s involvement in operation.

## Figures and Tables

**Table 1:** Comparison of IR and RF remotes [26,28].

	IR	RF
Frequency	870 nm and 930–950 nm	2.4 GHz Worldwide ISM-band
Carrier	38 KHz	315, 434, or 868 MHz
Coverage range	Up to 10 m	Up to 50 m
Data rate	10~1 K bit/s	100 K bit/s
Regulation	No limitations	Regulated in worldwide standards (ETSI, FCC, etc.)
Hardware cost	<$1	>$5
Objects or wall transmittance	Relatively little	High
Interference factor	Ambient light	Other communications (2.4 Ghz Wi-Fi, Bluetooth, etc.)

**Table 2:** IR protocols and keypad mapping according to the remotes.

	Protocol	Keypad Inputs	Raw_Data	Display on 7-Segment
Samsung TV	Samsung	On/Off	0xE0E040BF (32 bits)	1
		Keypad 1	0xE0E020DF (32 bits)	2
LG TV	NEC	On/Off	0x20DF10EF (32 bits)	3
		Keypad 1	0x20DF8877 (32 bits)	4
Set-top box	NEC	On/Off	0x01FE817E (32 bits)	5
		Keypad OK	0x01FEC837 (32 bits)	6

**Table 3:** Light transmittance measurement of curtain fabrics in Figure 11b.

	Fabric	Empty	①	②	③	④	⑤⑥
	
Lux		9800	2000	860	480	94	0
Light transmittance		100%	20.41%	8.78%	4.90%	0.96%	0%

## Data Availability

Not applicable.

## Supplementary Materials

The following is available online at https://youtu.be/VXxg-IvlqVU, Video S1: Eavesdropping Experiments.
